# The Potential Contribution of Fortified Maize Flour, Oil, Rice, Salt, and Wheat Flour to Estimated Average Requirements and Tolerable Upper Intake Levels for 15 Nutrients in 153 Countries

**DOI:** 10.3390/nu13020579

**Published:** 2021-02-09

**Authors:** Helena Pachón, Bethany Reynolds, Michelle Duong, Becky L. Tsang, Lana Childs, Corey L. Luthringer, Yunhee Kang, Florencia C. Vasta, Karen Codling

**Affiliations:** 1 Food Fortification Initiative, Atlanta, GA 30322, USA; lchilds44@gmail.com; 2 Hubert Department of Global Health, Emory University, Atlanta, GA 30322, USA; 3 Department of International Health, Johns Hopkins Bloomberg School of Public Health, Baltimore, MD 21205, USA; bethany.reynolds85@gmail.com (B.R.); ykang12@jhu.edu (Y.K.); 4 Iodine Global Network, P.O. Box 51030, 375 des Epinettes, Ottawa, ON K1E 3E6, Canada; mduong4@alumni.emory.edu (M.D.); btsang@ign.org (B.L.T.); corey.luthringer@gmail.com (C.L.L.); kcodling@ign.org (K.C.); 5 Global Alliance for Improved Nutrition, 1202 Geneva, Switzerland; fvasta@gainhealth.org

**Keywords:** enrichment, fortification, micronutrients, condiments, dietary reference intakes

## Abstract

Food fortification is designed to improve the nutritional profile of diets. The purpose of this research was to estimate the potential nutrient contribution of fortified maize flour, oil, rice, salt, and wheat flour in 153 countries, using the national intake (or availability) of the food and the nutrient levels required for fortification. This was done under two scenarios—maximum, where 100% of the food is assumed to be industrially processed and fortified, and realistic, where the maximum value is adjusted based on the percent of the food that is industrially processed and fortified. Under the maximum scenario, the median Estimated Average Requirements (EARs) met ranged from 22–75% for 14 nutrients (vitamins A, B1, B2, B3, B6, B12, D, E, folic acid and calcium, fluoride, iron, selenium and zinc), and 338% for iodine. In the realistic scenario, the median EARs met were 181% for iodine and <35% for the other nutrients. In both scenarios, the median Tolerable Upper Intake Levels (ULs) met were <55% for all nutrients. Under the realistic scenario, no country exceeded 100% of the UL for any nutrient. Current fortification practices of the five foods of interest have the global potential to contribute up to 15 nutrients to the diets of people, with minimal risk of exceeding ULs.

## 1. Introduction

Vitamin and mineral deficiencies are a global public health problem [1]. Because a basic cause of such micronutrient deficiencies is poverty, the prevalence of micronutrient deficiencies is greater in low- and middle-income countries than in high-income countries [2]. An estimated 51% of preschool children suffer from one or several micronutrient deficiencies [3]. In addition to children, women of childbearing age are at increased risk of suffering from micronutrient deficiencies [1]. Micronutrient deficiencies in these age groups affect child health outcomes including fetal and child growth, cognitive development, and function of the immune, nervous, and visual systems [4].

Iron, vitamin A and iodine deficiencies are the most common, according to the World Health Organization [5]. Generally considered the most prevalence micronutrient deficiency, iron deficiency can cause anemia in individuals of any age [6]. Iron deficiency during pregnancy can compromise the health of the woman, the fetus, and the newborn, as well as fetal and newborn neurological development. Work capacity can be diminished by iron deficiency. Because of its role in eye health, vitamin A deficiency is the leading cause of preventable child blindness [7]. Vitamin A deficiency can compromise immune function, increase an individual’s susceptibility to infections, and contribute to greater morbidity and mortality. Iodine deficiency disorders are the suite of health effects that iodine deficiency can provoke [8]. Iodine deficiency during pregnancy can cause goiter in women, and abortion, stillbirth, congenital anomalies, and mortality in the fetus. Children and adolescents who are iodine deficient can suffer impaired mental function and delayed physical development. Iodine deficiency in adults can result in reduced work productivity.

The immediate cause of micronutrient deficiencies is “insufficient intake [of micronutrients] or sufficient intakes combined with impaired absorption due to infection, disease, or inflammation” [2]. Food fortification is one strategy to prevent micronutrient deficiencies, by the addition of micronutrients to foods at the time of processing [1,9]. Virtually any processed condiments or foods can be enriched with one or more nutrients [1]. Condiments (hereafter referred to as foods) such as salt and fish sauce are commonly fortified with at least iodine and iron, respectively [10,11]. Staple foods such as wheat flour and milk are regularly fortified with B vitamins and fat-soluble vitamins, respectively [12,13]. Fortification requirements are codified in national standards, which specify, among others, the amount of nutrients to add to the fortified food [14].

Food fortification improves health outcomes and functional outcomes, as demonstrated in randomized controlled trials and evaluations of large-scale implementation in many countries. For example, fortification of salt with iodine (i.e., salt iodization) improves iodine status, thyroid function, child cognitive development, and congenital hypothyroidism [15]. Fortification of wheat and maize flour with folic acid improves folate status [16] and reduces the occurrence of birth defects of the brain and spine, such as spina bifida [17]. Surprisingly, few or no studies exist for the health impact of other nutrients added to fortified food such as vitamin B6 in maize flour, vitamin E in oil, and selenium in rice.

Estimating the potential dietary contribution of fortified foods is helpful in the planning and monitoring stages of national fortification programs [1]. In the former, such estimates help identify potential foods that are good vehicles for fortification and they establish expectations for the dietary impact that fortification is likely to have [18]. Once programs are implemented, monitoring dietary contribution serves several purposes. One is to determine if the anticipated dietary benefits are realized [1]. The answer to this question also determines if the timing is right to complete a costly biomarker evaluation of the fortification program [19]. Dietary contribution data also identify when nutrient levels in standards might need to be modified because they are either contributing too much or too little of the nutrient [20].

The Global Fortification Data Exchange (GFDx) is a website that visualizes diverse fortification data on maize flour, oil, rice, salt, and wheat flour, for up to 196 countries [21]. According to the GFDx, 153 countries mandate or allow the fortification of the aforementioned foods and have fortification requirements that include, at a minimum, the nutrient levels to add to the fortified food. Across fortification requirements for these five foods, there are up to fifteen micronutrients (singly or in combination) that are required or allowed to be added. For some foods, fortification adds more nutrients than what is naturally found in the food (e.g., iron in wheat flour) and for other foods, fortification adds nutrients that are not naturally found in the food (e.g., iodine in salt).

The goal of this study was to estimate the potential micronutrient contribution of fortified maize flour, oil, rice, salt, and wheat flour to the diet in countries with fortification requirements specified in standards. The analysis did not consider the naturally occurring nutrients in these foods.

## 2. Materials and Methods

Methodological details are provided in a thesis project report [22]. A summary is provided below.

### 2.1. Ethical Approval

The data used in this analysis are publicly available country-level data and do not provide information about specific individuals or groups. This study was deemed exempt from ethics review by the Institutional Review Boards at The Johns Hopkins University in April 2019 and Emory University in May 2019.

### 2.2. Study Design and Population

This is an ecological study using data from the Global Fortification Data Exchange (GFDx) from the 153 countries that specified nutrient levels to be added to maize flour, oil, rice, salt, or wheat flour, through mandatory or voluntary fortification, as of 7 December 2020 [21]. These foods were selected by the GFDx because they are among those most commonly fortified by a large number of countries or because international organizations regularly compile information on the fortification of these foods.

### 2.3. Variables

The following country- and food-specific data were drawn from the GFDx for the five foods of interest [21]—(1) fortification nutrient levels reported in milligrams per kilograms for all foods, (2) daily food intake expressed in grams per capita per day, (3) the food that is industrially processed is reported as a percentage, and (4) the food that is fortified is expressed as a percent.

The sources of the information varied. Fortification nutrient levels were extracted from the latest national standard. In those cases where two or more fortification compounds are included in standards for the same nutrient (e.g., potassium iodate and potassium iodide for iodine), the first compound in the GFDx database was selected. Daily food intake was extracted from published research for salt [23]; daily food availability was extracted from food balance sheets from the Food and Agriculture Organization of the United Nations for maize flour, oil, rice, and wheat flour [24]. The percentage of food that is industrially processed was obtained from various sources—country reports or surveys to fortification experts. The percent of food that was fortified was extracted from reports used for regulatory or informational purposes, or from food fortification experts.

Dietary reference intakes used in the analysis were as follows—Estimated Average Requirement (EAR) or a proxy and the Tolerable Upper Intake Level (UL) for non-pregnant, non-lactating adult females 19–50 years of age (Table S1).

- The EAR is the “nutrient intake value that is estimated to meet the requirements of half of healthy individuals in a group” [25]. It is the recommended indicator to estimate a population’s nutritional need [26], as well as fortification potential and safety [1]. The EAR was used for 13 of the 15 nutrients in the analysis. Since the EAR is lacking in fluoride, the Adequate Intake (AI) was used instead. The AI, which is conceptually more akin to a recommended dietary allowance than to an EAR, is “assumed to ensure nutritional adequacy” and in the case of fluoride, it is the amount “shown to maximize reductions in the incidence of dental caries without unwanted side effects, such as dental fluorosis” [27]. As fortifying with folic acid is recommended for the prevention of neural tube defects, the recommended intake of 400 µg of folic acid daily for women of reproductive age was used [28,29], instead of the lower EAR level, which is the amount recommended to normalize homocysteine levels. Lower doses of folic acid are not sufficient to prevent all folate-sensitive neural tube defects [30].
- The UL is “the highest level of daily nutrient intake that is likely to pose no risk of adverse health effects to almost all individuals in the general population” [25]; it refers to the chronic intake of nutrients. For the iodine UL, instead of the 1.1 mg/day value [25], a lower, 0.6 mg/day value was used [31]. The more conservative UL value was recommended as part of a harmonization exercise “to better identify any risk of potentially excessive intakes” [32].

### 2.4. Calculation of Potential Nutrient Contribution under Two Scenarios

The potential nutrient contribution was calculated for two scenarios for each country. The first was the maximum potential nutrient contribution (or, maximum) scenario—how much each nutrient could potentially contribute to diet if 100% of the food in the country was industrially processed and 100% was fortified according to fortification requirements (in standards). The second was a more realistic scenario that took into account, when available, the percentage of foods available in countries that are actually industrially processed and that are actually fortified.

**Figure 1:**
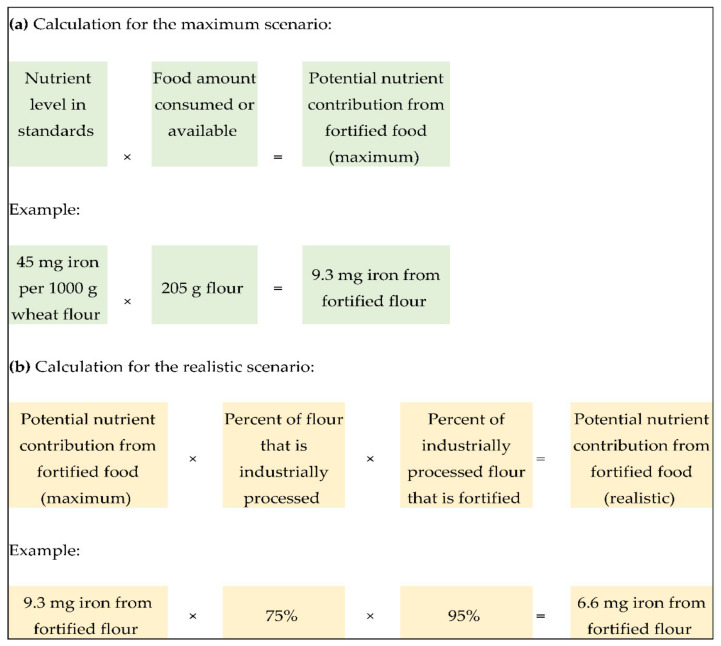
Calculation of potential nutrient contribution (in mg/capita/day) (**a**) under the maximum scenario where 100% of the food is assumed to be industrially processed and 100% is assumed to be fortified and (**b**) under the realistic scenario where the amount of the food that is industrially processed and fortified is known for a country.

For the first scenario, potential nutrient contribution (in mg/capita/day) was calculated by multiplying the fortification nutrient level (in mg/kg) by the daily food intake or availability (in grams/capita/day) and dividing by 1000 g (Figure 1). For the second, more realistic scenario, the value generated by the first scenario was multiplied by the percent of food industrially processed and by the percent of food fortified.

### 2.5. Comparison of Nutrient Contribution to Dietary Reference Intakes

The potential nutrient contribution of each micronutrient achieved through food fortification in the two scenarios was compared to nutrient-specific EAR and UL levels, to generate the percentage of the EAR and UL potentially met for each country. The number of countries that met <50%, 50–150%, or >150% of the EARs was calculated.

### 2.6. Limitations of the Analyses

The analyses suffer from limitations. First, the analyses only consider the nutrients added through fortification of maize flour, oil, rice, salt, and wheat flour. They do not consider the nutrient contribution of other foods in the diet including other fortified foods, supplements, or nutrients that are naturally found in the unfortified versions of the aforementioned five foods. Accordingly, the contribution of fortified maize flour, oil, rice, salt, and wheat flour to meeting nutrient gaps in the diet could not be estimated. Second, the intake or availability estimates for each food were based on one number per country (e.g., the amount of oil available for human consumption in Benin). These values were not calculated from surveys of hundreds or thousands of people, which would then allow for the calculation of the variability around mean or median intake. Ideally, the comparison of the potential fortification nutrient contribution to EARs and ULs is completed with such survey data that permits the calculation of the proportion of individuals with intakes below the EAR and above the UL. Such calculations could not be completed with the data presented in this study. The presentation of the median EARs and ULs met was to help with interpretation of the nutrient contribution values.

### 2.7. Descriptive Analyses

Using Microsoft Excel, descriptive analyses were performed on the potential nutrient contribution under both scenarios separately for all nutrients and for all foods combined.

## 3. Results

On 7 December 2020, the fortification requirements from national standards for maize flour, oil, rice, salt, or wheat flour, for 153 countries were accessed from the GFDx (Table 1). Most countries have fortification requirements for salt (*n* = 137), followed by wheat flour (*n* = 93). Among the nutrients, most countries have fortification requirements for iodine, followed by iron. The food-specific nutrients in fortification standards are noted in Table S2.

Under the maximum scenario, where 100% of the food is assumed to be industrially processed and 100% is assumed to be fortified according to national requirements, the median contribution of maize flour, oil, rice, salt, and wheat flour (combined) ranged from a low of 0.001 mg/c/d for vitamin B12 to a high of 204 mg/c/d for calcium (Table 2). When the contribution was compared to nutrient EARs, the median EAR percentage met among countries was less than 100% for all micronutrients. The notable exception was iodine where the median EAR met was 338%. When the contribution was compared to nutrient ULs, the median UL percentage met was less than 100% for all nutrients that had ULs, including iodine.

The number of countries meeting the nutrient-specific EARs was further broken down in Table 3. For most countries in the maximum scenario, the combination of up to five fortified foods could potentially contribute less than 50% of EARs for vitamins A, B2, B3, B12, D, E, folic acid, and calcium; between 50 and 150% of EARs for vitamins B1, B6, fluoride, iron and zinc; and more than 150% of the EAR for iodine. For the two countries with selenium in fortification requirements, one was classified as meeting less than 50% of EARs and the other as meeting 50–150% of EARs.

Table 4. For both values, the percentages were lower for maize flour and rice, highest for salt and wheat flour, and intermediate for oil.

As expected, under the realistic scenario where the percentage of the food that is industrially processed and fortified is known for a country (and is often less than 100%), the median contribution of fortified maize flour, oil, rice, salt, and wheat flour (combined) was lower for all nutrients than in the maximum scenario (Table 2). The median percent of EAR met among countries was less than 100% for all nutrients except iodine. When descriptive statistics were applied to the percent of UL met, the median percent was less than 100% for all nutrients, including iodine.

For most countries under the realistic scenario, the combination of up to five fortified foods could potentially contribute less than 50% of EARs for all nutrients, except iodine (Table 3). More than 150% of iodine EARs could be contributed by fortified salt in the majority of countries. Table S4 lists the potential nutrient contribution of maize flour, oil, rice, salt, and wheat flour (separately), when each is fortified according to national requirements in the maximum and realistic scenarios.

## 4. Discussion

If fortified to country requirements, maize flour, oil, rice, salt, and wheat flour (combined) can contribute up to 15 nutrients to the diet. Together, these fortified foods can contribute to reductions in vitamin and mineral deficiencies, without exposing the population to unduly high nutrient levels. The potential for nutrient contribution is greater when a higher proportion of the food is both industrially processed and actually fortified. In other words, country-level industry structure and compliance are decisive in determining whether the potential nutrient contribution from national fortification requirements is optimized. This analysis focused on the nutrients potentially contributed through the fortification of several foods. However, it excluded the contribution of nutrients from the rest of the diet (including non-fortified food, other fortified food, dietary supplements, and the nutrients naturally found in the fortified foods in the analysis).

In practice, most countries were estimated to meet 50% or less of EARs for 14 nutrients. If there are other micronutrient interventions in these countries, this might be an adequate amount to help reduce the nutrient gap between requirements and intake. For iodine, most countries’ fortification requirements could contribute more than 150% of the EAR. Given that the EAR is the amount needed to meet the nutritional needs of 50% of the population [26], 150% of the EAR is well below the UL. In the maximum scenario (where potential nutrient contribution was highest), the iodine contribution exceeded the UL for eight of 137 countries, ranging from 2–30% above the UL (Table S3). These findings suggest that the nutrient amounts potentially provided through maize flour, oil, rice, salt, and wheat flour fortification were safe.

There is a growing concern for the potential of nutrient interventions, especially when delivered simultaneously with other interventions in the same population, to contribute excessive nutrient levels [33]. The current analysis permitted evaluation of the simultaneous implementation of five fortified foods and in so doing found evidence in few countries that they would exceed ULs, a safe level to be consumed daily. This was not the case in Sri Lanka when several public health programs were analyzed for their joint contribution to micronutrient intakes [34]. The programs included vitamin A, iron, and iodine supplementation for preschool children, high-dose vitamin A supplementation for children and post-partum women, 15-nutrient micronutrient powders for children 6–23 months of age, food fortified with 15 nutrients and distributed through social protection programs to preschool children, mandatory salt fortification with iodine, and foods voluntarily fortified by industry with different nutrients. Among vitamin A, iron and iodine, the researchers identified vitamin A as the most concerning nutrient from an excess point of view. They concluded that “because the 95th percentile of current dietary exposure to vitamin A…was above the relevant UL, there is an appreciable risk of vitamin A toxicity”. The results from Sri Lanka highlight the importance of ongoing monitoring of all programs that deliver micronutrients to the population, including fortification.

In practice, a benefit–risk assessment is needed of the potential benefits and harms of fortification. Verhagen et al. [35] completed such an exercise for obligatory wheat flour fortification with folic acid in The Netherlands, a country that does not have this mandate. They modeled the number of people who stand to benefit from additional folic acid in the diet (e.g., due to a reduction in the incidence of neural tube defects and megaloblastic anemia) and those who stand to be harmed (e.g., due to an increase in the incidence of neurological effects from the masking of vitamin B12 deficiency and colorectal cancer), as well as the severity of these outcomes. They used these figures to estimate disability adjusted life years (DALYs). They concluded that the benefits of flour fortification with folic acid would outweigh the adverse effects as a net 7662 DALYs would be prevented in the country.

Several other studies assessed the nutrient contribution of fortified food alone; they focus on single countries [36], multiple countries [37], or have a global scope [38]. An example of each type is described to highlight methodological similarities, differences, advantages, and disadvantages compared to the current study.

Fieldler et al. converted food income and expenditure data from 10,000 households into apparent consumption by individuals for Bangladesh [36]. They estimated the nutrient gap in the whole diet (which is missing from the current analysis), as well as the potential contribution of fortified oil and wheat flour. While they only included the proportion of industrially processed foods in their analyses, they did not factor in the proportion that was fortified. The investigators compared potential intakes from fortified food against EARs but not ULs. For vitamin A fortification requirements of oil, they employed the 2006 fortification requirement (10–15 mg/kg), while the current analysis used the mid-point of the 2014 requirement (22.5 mg/kg). For wheat flour, the fortification requirements for vitamin A, iron, and zinc were the same in both analyses. At the national level, they estimated that fortified wheat flour and oil could together contribute 143 µg/day of vitamin A to the diet and fortified wheat flour could contribute 0.02 mg/day each of iron and zinc. Despite the differences between the datasets, assumptions and inputs into the analyses, the results were similar to the realistic scenario for the current analysis for the potential contribution of vitamin A from fortified oil and wheat flour (141 µg/day), iron from fortified wheat flour (0.02 mg/day) and zinc from fortified wheat flour (0.01 mg/day).

Friesen et al. converted food purchases from 1000 households each in Uganda and Tanzania to apparent intakes for individuals using nationally representative surveys [37]. Like the current analysis, they estimated potential nutrient contribution under two scenarios—maximum based on countries’ fortification requirements and realistic based on the amount of nutrients measured in food samples taken from households. The investigators used the Global Fortification Data Exchange as the source of fortification requirements. The GFDx derives this information from government-issued standards; the current analysis used the same source of information for fortification requirements. In both scenarios, only industrially processed foods were considered. Researchers estimated the potential contribution of fortified maize and wheat flour to iron and zinc intakes in both countries, fortified salt to iodine intakes in both countries, fortified oil to vitamin A intake in Tanzania; and fortified oil, maize flour, and wheat flour to vitamin A intake in Uganda. Similar to the current analysis, the proportion of women’s EARs met were calculated. Friesen et al. also calculated the proportion of women with intakes above ULs; this was not estimable in the current analysis.

The proportion of EARs potentially contributed by fortification varied between the Friesen study and current analysis [37]. Iron contributions to the EAR were not calculated for Tanzania and Uganda. Realistic scenarios were not calculable in the current analysis for vitamin A in Tanzania and for iodine in Uganda because of lack of data on the proportion of food industrially processed. For vitamin A in Tanzania in the maximum scenario, they calculated that fortified foods contributed 93.1% of EARs and the current analysis estimated that fortified foods contributed 171% of EARs. In Uganda, the realistic scenario yielded estimates that were approximately half those for the maximum scenario in both studies—53% maximum and 27% realistically by Friesen et a. and 164% maximum and 77% realistically in the current analysis. The iodine results for Tanzania were strikingly different—297% of EARs met in maximum scenario and 213% of EARs met in realistic scenario, as estimated by Friesen et al., as compared to 294% for maximum and 7% realistically in the current analysis. For Uganda, 377% of the iodine EAR was met in the maximum scenario, as calculated by Friesen et al.; the comparable estimate was 226% for the current analysis.

While the UL calculations were different between the current analysis and the study by Friesen et al., the conclusions were the same—there is a low risk of exceeding ULs for iron, vitamin A, and iodine [37]. In the maximum and realistic scenarios for iron and vitamin A in both countries, 0% of women had apparent intakes from fortified foods that exceeded the UL. Friesen et al. further estimated that 43% and <1% of women exceeded the iodine UL from fortified salt in both countries in the maximum and realistic scenarios, respectively. These results mirror those of the current analysis—in both scenarios, 0% of potential iron, vitamin A, and iodine contribution from fortified food exceeded the UL in both countries.

The closest research effort in geographic scope was by Smith et al. [38]. These researchers used FAO food balance sheet data to create a database with a supply of 23 nutrients from 225 foods, including some fortified foods in 152 countries. Similar to the current study, their database has one point estimate per country for the amount of each food available for human consumption. In terms of fortification, their databases includes a limited number of fortified foods (maize flour, margarine, milk, oil, sugar, rice, or wheat flour) and a limited number of nutrients (iron, zinc, and vitamins A, B1, B2, B3, or folic acid). In constructing the database for fortified wheat and maize flour, only the proportion of the flour that is industrially processed was considered. The independent contribution of fortification to nutrient supplies was not presented in the publication.

To summarize, there were several methodological advantages and disadvantages of the current study. The advantages were the large number of countries in the analysis. Among countries with mandatory or voluntary fortification legislation, the fortification requirements are known for the majority of them (GFDx 2020). The majority of countries with fortification requirements for maize flour (100%), oil (84%), rice (100%), salt (94%), and wheat flour (94%) were included in the analysis. The proxy for food intake used for each country stems from a uniform methodology implemented by FAO in all countries. Two factors that could reduce the potential nutrient contribution of fortified foods were assessed, when available, in the realistic scenario. The analysis considered fortification’s contributions to EARs and ULs.

The disadvantages included the use of one data point per country on food intake. Without distributions of intakes, further insights cannot be gleaned from calculating the proportion of individuals likely to benefit or at risk for harm from food fortification [26]. While a nationally representative salt intake figure was used in the analysis, for the other foods, a proxy of the amount available for human consumption was used. The limitations of this proxy are known [39]. When countries include multiple fortification compounds for the same nutrient (e.g., ferrous fumarate and NaFeEDTA for iron) in their requirements, only one was selected for the current analysis. In cases where the nutrient levels differed based on the fortification compound (e.g., 28 mg/kg for ferrous fumarate and 14 mg/kg for NaFeEDTA), the nutrient-contribution estimates differ if another compound is selected. For maize flour, oil, rice and wheat flour, the proportion industrially processed was estimated by fortification experts in the majority of countries; this information was not available for salt for any country. Similarly, for maize flour, rice, and wheat flour, the percent of these grains that were fortified were mainly drawn from fortification experts. Since these industry and compliance figures are not from official sources, such as government reports, they might differ from fortification practice in countries. In the absence of dietary intake information from the rest of the diet, including from other nutrition interventions, it is not possible to determine the proportion of the nutrient gap that is met by food fortification, or the percentage of individuals with nutrient intakes below EARs and above ULs.

How might country decision makers use the information in the current analysis? Decision makers might want to convene with private, public, and civic sector stakeholders to discuss the results presented here as well as other program-performance data for any of the following situations:

- If in the maximum scenario the potential nutrient contribution of any fortified food alone or in combination with others is substantially greater or lower than goals set for the country’s fortification program, then consider if the nutrient levels in standards might need to be changed.
- If the potential nutrient contribution is substantially different between the maximum and realistic scenarios, then discuss strategies for addressing any factors that are affecting program implementation. For example, how can compliance be improved if the percent of food that is fortified is not as high as it should be? If the percent of food that is industrially processed is low, discuss whether current fortification of this food is benefiting any subpopulations and whether other food vehicle options should be assessed to increase the reach of fortified foods.
- If the potential nutrient contribution in the maximum or realistic scenarios exceeds 100% of the UL, then consider if the nutrient levels in standards might need to be changed.
- If the potential nutrient contribution was not calculated for the realistic scenario, then seek information from industry partners and government regulatory authorities on the percent of food that is industrially processed and the percent of food that is fortified, respectively.
- Given the limitations of this analysis in only presenting the contribution of nutrients added through fortification, seek or convene studies that estimate the totality of the diet. In that context, is fortification performing as planned? Does the fortification program need to be adjusted?

## 5. Conclusions

This investigation used diverse data from most countries with fortification requirements for maize flour, oil, rice, salt, and wheat flour, to estimate their potential nutrient contribution. Current fortification practices for these foods have the potential to contribute up to 15 nutrients to the diet—some contribute a small proportion of nutrient requirements and others a greater percentage of requirements. In so doing, the fortified foods support country efforts to reduce and control micronutrient deficiencies with a food-based intervention. At the same time, there is little risk of these fortified foods, as designed and implemented, to contribute excess nutrients to the diet. Nevertheless, continuous monitoring of the nutrient contribution of fortified foods is warranted to ensure that effective and safe amounts of nutrients are delivered through fortification.

## Figures and Tables

**Table 1:** Countries, foods, and nutrients included in the analyses (GFDx 2020).

Countries with Fortification Requirements for ^1^	Number of Countries
Any food	153
Specific food ^2^	
Maize flour	19
Oil	32
Rice	14
Salt	137
Wheat flour	93
Specific nutrient ^3^	
Vitamin A	67
Vitamin B1 (thiamine)	98
Vitamin B2 (riboflavin)	86
Vitamin B3 (niacin)	95
Vitamin B6 (pyridoxine)	29
Vitamin B12	39
Vitamin D	22
Vitamin E	4
Folic acid	102
Calcium	28
Fluoride	20
Iodine	137
Iron	125
Selenium	2
Zinc	53

^1^ Countries where there are fortification requirements for any food (among maize flour, oil, rice, salt, or wheat flour), specific foods, or specific nutrients, as documented in national standards [21]. ^2^ The number of countries with fortification requirements for maize flour, oil, rice, salt, and wheat flour add up to more than 153 countries because two or more of these foods might have fortification requirements in a single country. ^3^ The number of countries with fortification requirements for the 15 nutrients add up to more than 153 countries because two or more nutrients might be required to be added to a single fortified food in a single country.

**Table 2:** Potential nutrient contribution under maximum and realistic scenarios expressed as median contribution (mg/capita/day), percent of Estimated of Average Requirement (EAR) met, and percent of Tolerable Upper Intake Level (UL) met for nutrients in fortification requirements for maize flour, oil, rice, salt, and wheat flour (combined).

Nutrient	In the Maximum Scenario ^1^, Median:				In the Realistic Scenario ^1^, Median:			
	*n*	Contribution ^2^ in mg/capita/day	% EAR Met ^3^	% UL Met ^4^	*n*	Median Contribution ^2^ in mg/capita/day	% EAR Met ^3^	% UL Met ^4^
Vitamin A	64	0.2	48.3	8.1	38	0.02	4.7	0.8
Vitamin B1 (thiamine)	92	0.7	75.4	-- ^5^	92	0.2	27.1	-- ^5^
Vitamin B2 (riboflavin)	81	0.4	46.1	-- ^5^	81	0.2	22.4	-- ^5^
Vitamin B3 (niacin)	89	5.4	49.2	15.5	89	1.9	17.0	5.4
Vitamin B6 (pyridoxine)	26	0.6	51.6	0.6	26	0.1	4.8	0.1
Vitamin B12	36	0.001	63.8	-- ^5^	36	0.0001	8.5	-- ^5^
Vitamin D	22	0.003	33.7	3.4	10	0.001	5.1	0.5
Vitamin E	4	2.6	21.9	0.3	1	2.3	19.6	0.2
Folic acid	96	0.2	49.7 ^6^	19.9	96	0.1	19.7 ^6^	7.9
Calcium	27	203.5	25.4	8.1	27	187.0	23.4	7.5
Fluoride	20	1.6	53.9 ^7^	16.2	1	0.9	31.5 ^7^	9.4
Iodine	136	0.3	338.3	53.6 ^8^	13	0.3	180.5	24.6 ^8^
Iron	117	5.2	63.6	11.5	115	2.2	27.0	4.9
Selenium	2	0.03	60.4	67.9	2	0.0002	20.6	2.3
Zinc	50	3.8	56.4	9.6	50	0.6	8.2	1.4

^1^ Maximum scenario where 100% of the food is assumed to be industrially processed and 100% is assumed to be fortified according to national standards and under the realistic scenario where, if the amount of the food that is industrially processed and fortified is known for a country, it is multiplied with the maximum scenario. ^2^ For the maximum scenario, potential nutrient contribution (in mg/capita/day) was calculated by multiplying the fortification nutrient level (in mg/kg) by the daily food intake or/availability (in grams/capita/day) and dividing by 1000 g). For the realistic scenario, the value generated by the maximum scenario was multiplied by the percent of food industrially processed and by the percent of food fortified, if these values are available for specific foods in specific countries. ^3^ For both scenarios, the potential nutrient contribution for each country was divided by the EAR for women of childbearing age and multiplied by 100. For example, for Afghanistan in the maximum scenario, the vitamin A contribution of all fortified foods was 0.093 mg/capita/day (Table S3). This value was divided by the vitamin A EAR (0.5 mg/day) (Table S1) and multiplied by 100, yielding 18.7% of the EAR. For all 64 countries with vitamin A fortification requirements, the median value of the % EAR was calculated and presented in Table 2. ^4^ For both scenarios, the potential nutrient contribution for each country was divided by the UL for women of childbearing age and multiplied by 100. If there is no UL for the nutrient, this figure was not calculated. For example, for Afghanistan in the maximum scenario, the vitamin A contribution of all fortified foods was 0.093 mg/capita/day (Table S3). This value was divided by the vitamin A UL (3 mg/day) (Table S1) and multiplied by 100, yielding 3.1% of the UL. For all 64 countries with vitamin A fortification requirements, the median value of the % UL was calculated and presented in Table 2. ^5^ Due to the absence of suitable data for vitamins B1, B2, and B12, no UL for these micronutrients can be established [25]. Thus, the proportion of ULs met cannot be calculated for these nutrients. ^6^ For folic acid, the EAR was not used. Because fortifying with folic acid is recommended for the prevention of neural tube defects, the recommended intake of 400 µg of folic acid daily for women of reproductive age was used [28,29] instead of the lower EAR level, which is the amount recommended to normalize homocysteine levels. Lower doses of folic acid are not sufficient to prevent all folate-sensitive neural tube defects [30]. ^7^ The Adequate Intake (AI) level was used for fluoride, as an EAR is not set for this nutrient [25]. ^8^ For the iodine UL, instead of the 1.1 mg/day value from the National Academies of Sciences, Engineering, and Medicine, a lower, 0.6 mg/day value was used [31]. The more conservative UL value was recommended as part of a harmonization exercise [32].

**Table 3:** The number of countries where the potential nutrient contribution under maximum and realistic scenarios was less than 50%, between 50 and 150% or greater than 150% of the Estimated of Average Requirement (EAR) met, when the nutrients in fortification requirements for maize flour, oil, rice, salt, and wheat flour are combined.

Nutrient	Maximum Scenario ^1,2^								Realistic Scenario ^1,2^							
	<50% Met		50–150% Met		>150% Met		Total		<50% Met		50–150% Met		>150% Met		Total	
	*n*	%	*n*	%	*n*	%	*n*	%	*n*	%	*n*	%	*n*	%	*n*	%
Vitamin A	32	50.0	26	40.6	6	9.4	64	100.0	32	84.2	6	15.8	0	0.0	38	100.0
Vitamin B1(thiamine)	28	30.4	49	53.3	15	16.3	92	100.0	56	60.9	29	31.5	7	7.6	92	100.0
Vitamin B2 (riboflavin)	44	54.3	36	44.4	1	1.2	81	100.0	62	76.5	19	23.5	0	0.0	81	100.0
Vitamin B3 (niacin)	46	51.7	40	44.9	3	3.4	89	100.0	66	74.2	23	25.8	0	0.0	89	100.0
Vitamin B6 (pyridoxine)	12	46.2	13	50.0	1	3.8	26	100.0	23	88.5	3	11.5	0	0.0	26	100.0
Vitamin B12	16	44.4	13	36.1	7	19.4	36	100.0	31	86.1	5	13.9	0	0.0	36	100.0
Vitamin D	16	72.7	4	18.2	2	9.1	22	100.0	10	100.0	0	0.0	0	0.0	10	100.0
Vitamin E	4	100.0	0	0.0	0	0.0	4	100.0	1	100.0	0	0.0	0	0.0	1	100.0
Folic acid ^3^	48	50.0	40	41.7	8	8.3	96	100.0	70	72.9	24	25.0	2	2.1	96	100.0
Calcium	20	74.1	7	25.9	0	0.0	27	100.0	22	81.5	5	18.5	0	0.0	27	100.0
Fluoride ^4^	7	35.0	12	60.0	1	5.0	20	100.0	1	100.0	0	0.0	0	0.0	1	100.0
Iodine	0	0.0	3	2.2	133	97.8	136	100.0	2	15.4	2	15.4	9	69.2	13	100.0
Iron	43	36.8	60	51.3	14	12.0	117	100.0	72	62.6	38	33.0	5	4.3	115	100.0
Selenium	1	50.0	1	50.0	0	0.0	2	100.0	2	100.0	0	0.0	0	0.0	2	100.0
Zinc	20	40.0	22	44.0	8	16.0	50	100.0	43	86.0	6	12.0	1	2.0	50	100.0

^1^ Maximum scenario where 100% of the food is assumed to be industrially processed and 100% is assumed to be fortified according to national standards and under the realistic scenario where, if the amount of the food that is industrially processed and fortified is known for a country, it is multiplied with the maximum scenario. ^2^ For the maximum scenario, potential nutrient contribution (in mg/capita/day) was calculated by multiplying the fortification nutrient level (in mg/kg) by the daily food intake or/availability (in grams/capita/day) and dividing by 1000 g. For the realistic scenario, the value generated by the maximum scenario was multiplied by the percent of food industrially processed and by the percent of food fortified, if these values are available for specific foods in specific countries. For both scenarios, the potential nutrient contribution was divided by the EAR for women of childbearing age (Table S1) and multiplied by 100. ^3^ For folic acid, the EAR was not used. Because fortifying with folic acid is recommended for the prevention of neural tube defects, the recommended intake of 400 µg of folic acid daily for women of reproductive age was used [28,29] instead of the lower EAR level, which is the amount recommended to normalize homocysteine levels. Lower doses of folic acid are not sufficient to prevent all folate-sensitive neural tube defects [30]. ^4^ The Adequate Intake (AI) level was used for fluoride, as an EAR is not set for this nutrient [25].

**Table 4:** The percentage of foods industrially processed ^1^ and the percentage of foods fortified ^2^ in countries with these data (GFDx 2020).

Food	Industrially Processed		Fortified	
	*n* ^3^	Median (%)	*n* ^3^	Median (%)
Maize flour	19	20.0	19	5.0
Oil	5	85.0	11	59.7
Rice	14	39.5	14	0.1
Salt	25	100.0	34	77.1
Wheat flour	93	100.0	93	95.0

^1^ The proportion of foods that are produced in industrial settings (versus cottage industries). ^2^ The proportion of foods that are fortified; might also be called fortification compliance. ^3^ The number of countries with these data.

## Data Availability

Data are available at the Global Fortification Data Exchange: FortificationData.org.

## Supplementary Materials

The following are available online at https://www.mdpi.com/2072-6643/13/2/579/s1. Table S1: The Estimated Average Requirements (EARs) and Tolerable Upper Intake Levels (ULs) for nutrients in the analysis [25], non-pregnant, non-lactating adult females 19–50 years of age. Table S2: Number of countries with specific nutrients in standards for food fortification. Table S3: The potential nutrient contribution of maize flour, oil, rice, salt, and wheat flour (separately) fortified according to national requirements in the maximum and realistic scenarios, by country, for 153 countries. Table S4: A summary of the potential nutrient contribution of maize flour, oil, rice, salt, and wheat flour (separately) fortified according to national requirements in the maximum and realistic scenarios for 153 countries.
