# Supplementary material for: The Potential Contribution of Fortified Maize Flour, Oil, Rice, Salt, and Wheat Flour to Estimated Average Requirements and Tolerable Upper Intake Levels for 15 Nutrients in 153 Countries
Source: Nutrients. 2021 Feb 9;13(2):579. doi: 10.3390/nu13020579 (PMC7916358; doi:10.3390/nu13020579)
Supplement: Supplementary file 1 [file nutrients-13-00579-s001.zip › Tables S1_S2_S4_8 Feb 2021.docx]

**Table S1.** The Estimated Average Requirements (EARs) and Tolerable Upper Intake Levels (ULs) for nutrients in the analysis [25], non-pregnant, non-lactating adult females 19-50 years of age.

| **Nutrient** | **Estimated Average Requirement (mg/day)** | **Tolerable Upper Intake Level (mg/day)** |
| --- | --- | --- |
| Vitamin A | 0.5 | 3 |
| Vitamin B1 (thiamine)^1^ | 0.9 | Not determined |
| Vitamin B2 (riboflavin)^1^ | 0.9 | Not determined |
| Vitamin B3 (niacin) | 11 | 35 |
| Vitamin B6 (pyridoxine) | 1.1 | 100 |
| Vitamin B12^1^ | 0.002 | Not determined |
| Vitamin D | 0.01 | 0.1 |
| Vitamin E | 12 | 1000 |
| Folic acid^2^ | 0.4 | 1 |
| Calcium | 800 | 2500 |
| Fluoride^3^ | 3 | 10 |
| Iodine^4^ | 0.095 | 0.6 |
| Iron | 8.1 | 45 |
| Selenium | 0.045 | 0.4 |
| Zinc | 6.8 | 40 |

^1^ Due to the absence of suitable data for vitamins B1, B2, and B12, no UL for these micronutrients can be established [25]. Thus, the proportion of ULs met cannot be calculated for these nutrients.

^2^For folic acid, the EAR was not used.  Because fortifying with folic acid is recommended for the prevention of neural tube defects, the recommended intake of 400 µg of folic acid daily for women of reproductive age was used [28,29] instead of the lower EAR level, which is the amount recommended to normalize homocysteine levels. Lower doses of folic acid are not sufficient to prevent all folate-sensitive neural tube defects [30].

^3^The EARs were used for all micronutrients with the exception of fluoride. The Adequate Intake (AI) level was used for fluoride, as an EAR is not set for this nutrient [25].

^4^For the iodine UL, instead of the 1.1 mg/day value from the National Academies of Sciences, Engineering, and Medicine, a lower, 0.6 mg/day value was used [31]. The more conservative UL value was recommended as part of a harmonization exercise [32].

**Table S2.** Number of countries with specific nutrients in standards for food fortification [21].

| **Countries with fortification requirements for^1^** | **Number of countries^2^** |
| --- | --- |
| Maize flour |  |
| Vitamin A | 11 |
| Vitamin B1 (thiamine) | 17 |
| Vitamin B2 (riboflavin) | 16 |
| Vitamin B3 (niacin) | 17 |
| Vitamin B6 (pyridoxine) | 8 |
| Vitamin B12 | 10 |
| Vitamin D | 1 |
| Folic acid | 17 |
| Calcium | 2 |
| Iron | 19 |
| Zinc | 12 |
| Oil |  |
| Vitamin A | 32 |
| Vitamin D | 13 |
| Vitamin E | 3 |
| Rice |  |
| Vitamin A | 4 |
| Vitamin B1 (thiamine) | 13 |
| Vitamin B2 (riboflavin) | 4 |
| Vitamin B3 (niacin) | 12 |
| Vitamin B6 (pyridoxine) | 5 |
| Vitamin B12 | 7 |
| Vitamin D | 3 |
| Vitamin E | 1 |
| Folic acid | 10 |
| Calcium | 2 |
| Iron | 13 |
| Selenium | 1 |
| Zinc | 8 |
| Salt |  |
| Fluoride | 20 |
| Iodine | 137 |
| Iron | 2 |
| Wheat flour |  |
| Vitamin A | 22 |
| Vitamin B1 (thiamine) | 68 |
| Vitamin B2 (riboflavin) | 66 |
| Vitamin B3 (niacin) | 66 |
| Vitamin B6 (pyridoxine) | 16 |
| Vitamin B12 | 22 |
| Vitamin D | 7 |
| Folic acid | 75 |
| Calcium | 24 |
| Iron | 91 |
| Selenium | 1 |
| Zinc | 33 |

^1^Countries where there are fortification requirements for a specific food, as documented in national standards [21].

^2^For each food, the number of countries with fortification requirements for maize flour, oil, rice, salt, and wheat flour add up to more than 19, 32, 14, 137 and 93 countries, respectively, because two or more nutrients may be required to be added to the fortified food in a country.

**Table S4.** A summary of the potential nutrient contribution of maize flour, oil, rice, salt, and wheat flour (separately) fortified according to national requirements in the maximum and realistic scenarios for 153 countries [21].

|  | **In the Maximum Scenario^1^, Median:** | | | | **In the Realistic Scenario^1^, Median:** | | | |
| --- | --- | --- | --- | --- | --- | --- | --- | --- |
| **Nutrient** | **n** | **Contribution^2^ in mg/capita/day** | **% EAR met^3^** | **% UL met^4^** | **n** | **Median contribution^2^ in mg/capita/day** | **% EAR met^3^** | **% UL met^4^** |
| *Maize Flour* | | | | | | | | |
| Vitamin A | 10 | 0.2 | 37.1 | 6.2 | 10 | 0.01 | 1.4 | 0.2 |
| Vitamin B1 (thiamine) | 16 | 0.6 | 68.5 | --^5^ | 16 | 0.02 | 2.6 | --^5^ |
| Vitamin B2 (riboflavin) | 15 | 0.5 | 53.1 | --^5^ | 15 | 0.02 | 1.9 | --^5^ |
| Vitamin B3 (niacin) | 16 | 7.2 | 65.6 | 20.6 | 16 | 0.1 | 1.3 | 0.4 |
| Vitamin B6 (pyridoxine) | 7 | 0.9 | 80.5 | 0.9 | 7 | 0.03 | 2.6 | 0.03 |
| Vitamin B12 | 9 | 0.001 | 54.2 | --^5^ | 9 | 0.00 | 3.8 | --^5^ |
| Vitamin D | 1 | 0.001 | 11.3 | 1.1 | 1 | 0.00 | 3.3 | 0.3 |
| Folic acid | 16 | 0.3 | 63.2^6^ | 25.3 | 16 | 0.01 | 2.0^6^ | 0.8 |
| Calcium | 2 | 259.9 | 32.5 | 10.4 | 2 | 6.6 | 0.8 | 0.3 |
| Iron | 18 | 4.0 | 49.2 | 8.9 | 18 | 0.1 | 1.3 | 0.2 |
| Zinc | 11 | 4.2 | 62.5 | 10.6 | 11 | 0.2 | 3.4 | 0.6 |
|  |  |  |  |  |  |  |  |  |
| *Oil* | | | | | | | | |
| Vitamin A | 28 | 0.3 | 66.3 | 11.1 | 4 | 0.1 | 27.9 | 4.7 |
| Vitamin D | 11 | 0.00 | 31.2 | 3.1 | 1 | 0.001 | 4.8 | 0.5 |
| Vitamin E | 3 | 2.9 | 24.3 | 0.3 | 0 | --^9^ | --^9^ | --^9^ |
|  |  |  |  |  |  |  |  |  |
| *Rice* | | | | | | | | |
| Vitamin A | 4 | 0.6 | 113.7 | 19.0 | 4 | 0.0002 | 0.03 | 0.01 |
| Vitamin B1 (thiamine) | 11 | 0.9 | 102.4 | --^5^ | 11 | 0 | 0 | --^5^ |
| Vitamin B2 (riboflavin) | 3 | 0.4 | 47.1 | --^5^ | 3 | 0 | 0 | --^5^ |
| Vitamin B3 (niacin) | 10 | 8.0 | 72.3 | 22.7 | 10 | 0 | 0 | 0 |
| Vitamin B6 (pyridoxine) | 5 | 0.8 | 68.5 | 0.8 | 5 | 0 | 0 | 0 |
| Vitamin B12 | 7 | 0.002 | 94.2 | --^5^ | 7 | 0 | 0.02 | --^5^ |
| Vitamin D | 3 | 0.002 | 17.3 | 1.7 | 3 | 0 | 0 | 0 |
| Vitamin E | 1 | 2.3 | 19.6 | 0.2 | 1 | 2.3 | 19.6 | 0.2 |
| Folic acid | 10 | 0.3 | 66.4^6^ | 26.5 | 10 | 0 | 0.004^6^ | 0.002 |
| Calcium | 2 | 60.3 | 7.5 | 2.4 | 2 | 9.7 | 1.2 | 0.4 |
| Iron | 11 | 10.0 | 123.1 | 22.2 | 11 | 0 | 0 | 0 |
| Selenium | 1 | 0.02 | 40.6 | 4.6 | 1 | 0 | 40.6 | 4.6 |
| Zinc | 8 | 7.5 | 110.9 | 18.8 | 8 | 0.002 | 0.03 | 0.005 |
|  |  |  |  |  |  |  |  |  |
| *Salt* | | | | | | | | |
| Fluoride | 20 | 1.6 | 53.9^7^ | 16.2 | 1 | 0.9 | 31.5^7^ | 9.4 |
| Iodine | 136 | 0.3 | 338.3 | 53.6^8^ | 13 | 0.3 | 180.5 | 24.6^8^ |
| Iron | 2 | 4.6 | 57.3 | 10.3 | 0 | --^9^ | --^9^ | --^9^ |
|  |  |  |  |  |  |  |  |  |
| *Wheat flour* | | | | | | | | |
| Vitamin A | 20 | 0.1 | 29.3 | 4.9 | 20 | 0.0 | 9.4 | 1.6 |
| Vitamin B1 (thiamine) | 65 | 0.7 | 73.0 | --^5^ | 65 | 0.5 | 53.9 | --^5^ |
| Vitamin B2 (riboflavin) | 63 | 0.4 | 44.2 | --^5^ | 63 | 0.3 | 35.5 | --^5^ |
| Vitamin B3 (niacin) | 63 | 5.1 | 46.2 | 14.5 | 63 | 3.9 | 35.2 | 11.1 |
| Vitamin B6 (pyridoxine) | 14 | 0.4 | 35.3 | 0.4 | 14 | 0.2 | 16.4 | 0.2 |
| Vitamin B12 | 20 | 0.001 | 46.2 | --^5^ | 20 | 0.0004 | 26.2 | --^5^ |
| Vitamin D | 5 | 0.004 | 38.4 | 3.8 | 5 | 0.004 | 36.4 | 3.6 |
| Folic acid | 70 | 0.2 | 47.1^6^ | 18.8 | 70 | 0.1 | 34.1^6^ | 13.6 |
| Calcium | 23 | 205.4 | 25.7 | 8.2 | 23 | 203.5 | 25.4 | 8.1 |
| Iron | 86 | 5.1 | 62.9 | 11.3 | 86 | 4.0 | 49.2 | 8.9 |
| Selenium | 1 | 0.04 | 80.2 | 9.0 | 1 | 0.0003 | 0.7 | 0.1 |
| Zinc | 31 | 2.9 | 41.9 | 7.1 | 31 | 1.8 | 26.0 | 4.4 |

^1^ Maximum scenario where 100% of the food is assumed to be industrially processed and 100% is assumed to be fortified according to national standards and **(b)** under the realistic scenario where, if the amount of the food that is industrially processed and fortified is known for a country, it is multiplied with the maximum scenario.

^2^ For the maximum scenario, potential nutrient contribution (in mg/capita/day) was calculated by multiplying the fortification nutrient level (in mg/kg) by the daily food intake or /availability (in grams/capita/day) and dividing by 1,000 grams). For the realistic scenario, the value generated by the maximum scenario was multiplied by the percent of food industrially processed and by the percent of food fortified, if these values are available for specific foods in specific countries.

^3^ For both scenarios, the potential nutrient contribution for each country and food was divided by the EAR for women of childbearing age and multiplied by 100. For example, for Zimbabwe in the maximum scenario, the iodine contribution of fortified salt was 0.315 mg/capita/day (Table S3). This value was divided by the iodine EAR (0.095 mg/day) (Table S1) and multiplied by 100, yielding 332% of the EAR. For all 136 countries with salt iodization, the median value of the % EAR was calculated and presented in Table S4.

^4^ For both scenarios, the potential nutrient contribution for each country and food was divided by the UL for women of childbearing age and multiplied by 100. If there is no UL for the nutrient, this figure was not calculated. For example, for Zimbabwe in the maximum scenario, the iodine contribution of fortified salt was 0.315 mg/capita/day (Table S3). This value was divided by the iodine UL (0.6 mg/day) (Table S1) and multiplied by 100, yielding 53% of the UL. For all 136 countries with salt iodization requirements, the median value of the % UL was calculated and presented in Table S4.

^5^ Due to the absence of suitable data for vitamins B1, B2, and B12, no UL for these micronutrients can be established [25]. Thus, the proportion of ULs met cannot be calculated for these nutrients.

^6^For folic acid, the EAR was not used.  Because fortifying with folic acid is recommended for the prevention of neural tube defects, the recommended intake of 400 µg of folic acid daily for women of reproductive age was used [28,29] instead of the lower EAR level, which is the amount recommended to normalize homocysteine levels. Lower doses of folic acid are not sufficient to prevent all folate-sensitive neural tube defects [30].

^7^ The Adequate Intake (AI) level was used for fluoride, as an EAR is not set for this nutrient [25].

^8^For the iodine UL, instead of the 1.1 mg/day value from the National Academies of Sciences, Engineering, and Medicine, a lower, 0.6 mg/day value was used [31]. The more conservative UL value was recommended as part of a harmonization exercise [32].

^9^No countries had data for this scenario; this value could not be calculated [31].
